# Supplementary material for: Lignin-Modifying Enzymes in Scedosporium Species
Source: J Fungi (Basel). 2023 Jan 12;9(1):105. doi: 10.3390/jof9010105 (PMC9861984; doi:10.3390/jof9010105)
Supplement: Supplementary file 1 [file jof-09-00105-s001.zip › jof-2087755-SI.pdf]

Table S1. Primers used in this study.

Gene (Accession CDS number)	Primer sequence
**Peroxidases**	
Cytochrome c peroxidase (SAPIO_CDS3675)	1F : AAG TGG CAG TGG AGG AAG TG<br>1R : CGG AAC TCC TTG TCC TGG AC
Beta-1,3 exoglucanase (SAPIO_CDS10447)	2F : CGC TGG ATA AGT GTG CGG A<br>2R : TGT TGC AGT TGG AGA TGG GG<br>3F : GGC TTC GAT GAC TCT TTT GC<br>3R : GTT TGC TGC TTC TGG AGG AC
Catalase peroxidase (SAPIO_CDS10583)	4F : TAT CGT GTC TTC GAC GGT CG<br>4R : AAA CAT TGT CGG GCC AGC TA<br>5F : CAC GCA TCT GGA ACA TAT CG<br>5R : AGT AGA TCC GCC CAG GAG AT
Pseudogene (SAPIO_CDS4198)	6F : CTC AAC AGC TGG CCT GAC AA<br>6R : CTC TCG GCA CCC CAA TAG AC
**Oxidases**	
Pseudogene (SAPIO_CDS2438)	7F : GAG GAC GCA ATA CTG ACA AGC<br>7R : TGT TAG ATC CAC CAC GTC GC
Iron transport multicopper oxidase (SAPIO_CDS0314)	8F : ACC GGT GGT ACC AGT CAG AA<br>8R : TTT ACC GTC GGC TGG GTT AC
Iron transport multicopper oxidase (SAPIO_CDS8659)	9F : GCC TGC GGA TAA GAC TTT GGT<br>9R : GTC TCG TTG GTA TTG AAG TTA CC<br>10F : TCG GGG TAT TGT GCC ATA TT<br>10R : CCA CCT GGC GAG AGT ATT GT
L-ascorbate (SAPIO_CDS10367)	11F : GAT GGT GTC CAT GGC GTG A<br>11R : TCG TGG AAG TGA TCA CCG AAC
Hypothetical protein (SAPIO_CDS2597)	12F : ACG GCA TCA GGC AGA TTG<br>12R : CCA CTG CAG GGC CAT TTA
Laccase (SAPIO_CDS6544)	13F : GGA ATG GTG CGA CTT TTC CG<br>13R : CCG TGA AAG TGA AGG CTG GT<br>14F : GGC AAT AAC CGT CAA GGT GT<br>14R : GTG GTA CCA TTC CCA TTT CG
Uncharacterized protein (SAPIO_CDS9845)	15F : ATG ATG GTG CCA ATG GTG TCA<br>15R : GGT GCC AAG AAG ACC GTT
Endo-α-N-acetylgalactosaminidase (SAPIO_CDS0469)	16F : CTT CCG CAT TGG CCT TGA TG<br>16R : ACT TCG ATG ATT TGG CCC TT
Iron transport multicopper oxidase (SAPIO_CDS0322)	17F : GGC CAA CGA TGC AGT TTC<br>17R : CAA CTC GGG TGG TAG AAC ATC A
Hypothetical protein (SAPIO_CDS8589)	18F : GAA TCA CAA GCT GCA TCC CG<br>18R : CCC ATC CCA TCC ACA AGC AC
Pseudogene (SAPIO_CDS4646)	19F : ACA TCA GTC CGT ACA TGC CA<br>19R : GAC GAA TCG AGA CGA GAC GG

**Reference genes**	
Transcription protein on Pol III promoters – *TFC1* (SAPIO_CDS10483)	20F: CAT CCA CCG TGC GTG GCA AA<br>20R: CAT CAT CAA TCC TCG AGG GC
Ubiquitin carrier protein – *UbcB* (SAPIO_CDS7304)	21F: CCG GAG AGT TGT GCC TTG A<br>21R: CAG GTT CGA TGC TTC GAC G
	22F: GGC ACC TTT AGG CTG GTA AT<br>22R: GCC GGT GTT TGG ATC GTT TA
Rho GTPase activator – *Sac7* (SAPIO_CDS7786)	23F: CCG ATC GGT TGG ATT AGG AAC<br>23R: CAA GAC TCT TGC CAC GAA GG
	24F: GCT ATG TCC CAA TCG TCG TG<br>24R: CTT TGC CGT ATC TGT CAG GC
Mitochondrial membrane fission protein – *Fis1* (SAPIO_CDS7496)	25F: GAT CAG TTC GAG AAG GAG GG<br>25R: GGA AGA TGT CGG AGA GGA GA
	26F: TTC GAG AAG GAG GGT GAG AT<br>26R: GTA GTT GCC CAG CTT GTA GTT
GTP-binding protein – *SarA* (SAPIO_CDS5864)	27F: CAT CAG CTC GGT CTC TAC CA<br>27R: CGT ATT GGG ACA ACC ACC GT
Protein catabolic process – *UBC6* (SAPIO_CDS4591)	28F: GAG AAC CCG CCA CCA TAC A<br>28R: CAA TAC TGG CCG CCA TGG TA
	29F: GCC TTG ACA TTG CCC TTG AG<br>29R: TGC CTT TCC ATC TCC GAC TT
Actin (SAPIO_CDS9600)	30F: CCC TTG ACT TTG AGC AGG AGA TCC A<br>30R: CTC AAG ACC GAG GAC AGA GGG CTG
Beta-tubulin – *Tub* (SAPIO_CDS5417)	31F: TTC CGC AAC GGC CGA TAC CT<br>31R: CCA CGG GGA GGA ATA GAG CA
GAPDH (SAPIO_CDS3929)	32F: TCA ACG GTT TCG GTC GTA TC<br>32R: GCG TAC TTG GTC TCG ATG AA

Table S2. PCR efficiency according to *Scedosporium* species and the primer pair used.

Gene (Accesion CDS number)	***S. apiospermum***		***S. aurantiacum***		***S. dehoogii***	
	Primer pair	Efficiency	Primer pair	Efficiency	Primer pair	Efficiency
**Peroxidases**						
Cytochrome c peroxidase (SAPIO_CDS3675)	1	113%	1	110%	1	95%
Beta-1,3 exoglucanase (SAPIO_CDS10447)	2	109%	2	102%	3	89%
Catalase peroxidase (SAPIO_CDS10583)	4	97%	4	102%	5	87%
Pseudogene (SAPIO_CDS4198)	6	102%	6	119%	6	91%
**Oxidases**						
Pseudogene (SAPIO_CDS2438)	7	107%	7	104%	7	92%
Iron transport multicopper oxidase (SAPIO_CDS0314)	8	84%	8	106%	8	81%
Iron transport multicopper oxidase (SAPIO_CDS8659)	9	97%	9	108%	10	81%
L-ascorbate (SAPIO_CDS10367)	11	96%	11	103%	11	91%
Hypothetical protein (SAPIO_CDS2597)	12	102%	12	94%	12	91%
Laccase (SAPIO_CDS6544)	13	102%	13	100%	14	85%
Uncharacterized protein (SAPIO_CDS9845)	15	106%	15	110%	15	102%
Endo-α-N-acetylgalactosaminidase (SAPIO_CDS0469)	16	92%	16	113%	16	98%
Iron transport multicopper oxidase (SAPIO_CDS0322)	17	96%	17	114%	17	102%
Hypothetical protein (SAPIO_CDS8589)	18	87%	18	107%	18	89%
Pseudogene (SAPIO_CDS4646)	19	98%	19	107%	19	92%
**Reference genes**						
Transcription protein on Pol III promoters – *TFC1* (SAPIO_CDS10483)	20	95%	20	97%	20	97%
Ubiquitin carrier protein – *UbcB* (SAPIO_CDS7304)	21	91%	22	101%	21	106%
Rho GTPase activator – *Sac7* (SAPIO_CDS7786)	23	102%	24	76%	23	95%
Mitochondrial membrane fission protein – *Fis1* (SAPIO_CDS7496)	25	91%	26	102%	25	97%
GTP-binding protein – *SarA* (SAPIO_CDS5864)	27	96%	27	107%	27	88%
Protein catabolic process – *UBC6* (SAPIO_CDS4591)	28	96%	29	95%	28	85%
Actin (SAPIO_CDS9600)	30	99%	30	110%	30	86%
Beta-tubulin – *Tub* (SAPIO_CDS5417)	31	100%	31	97%	31	88%
GAPDH (SAPIO_CDS3929)	32	81%	32	99%	32	87%

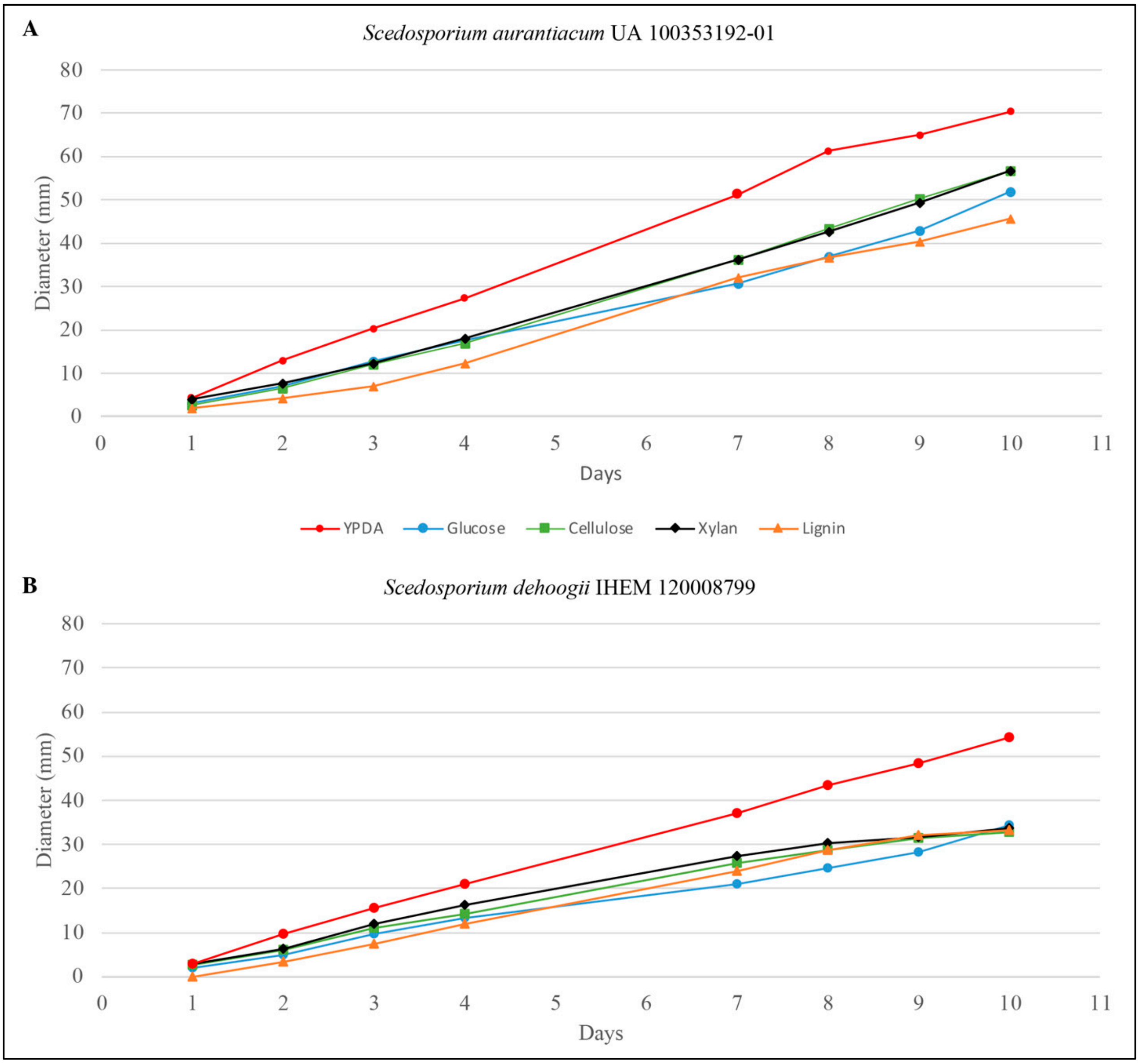

Figure S1. Kinetics of growth of *Scedosporium aurantiacum* UA 100353192-01 **(A)** and *Scedosporium dehoogii* IHEM 120008799 **(B)** on a synthetic agar-based medium Scedo-Select III containing a unique carbon source: glucose for control conditions, lignin, cellulose or xylan.
